# Effect of bubble size on ultrasound backscatter from bubble clouds in the context of gas kick detection in boreholes

**DOI:** 10.1038/s41598-023-38937-6

**Published:** 2023-07-21

**Authors:** Shivanandan Indimath, Stefano Fiorentini, Bjarne Rosvoll Bøklepp, Jørgen Avdal, Tonni Franke Johansen, Svein-Erik Måsøy

**Affiliations:** 1 Norwegian University of Science and Technology, Center for Innovative Ultrasound Solutions, 7491 Trondheim, Norway; 2 Equinor ASA, TDI EDT EDP Data Management Architecture and Analytics, 7053 Trondheim, Norway; 3 Sintef Digital, 7034 Trondheim, Norway

**Keywords:** Acoustics, Crude oil, Natural gas, Carbon capture and storage, Geothermal energy

## Abstract

Early detection of gas influx in boreholes while drilling is of significant interest to drilling operators. Several studies suggest a good correlation between ultrasound backscatter/attenuation and gas volume fraction (GVF) in drilling muds, and thereby propose methods for quantification of GVF in boreholes. However, the aforementioned studies neglect the influence of bubble size, which can vary significantly over time. This paper proposes a model to combine existing theories for ultrasound backscatter from bubbles depending on their size, viz. Rayleigh scattering for smaller bubbles, and specular reflection for larger bubbles. The proposed model is demonstrated using simulations and experiments, where the ultrasound backscatter is evaluated from bubble clouds of varying bubbles sizes. It is shown that the size and number of bubbles strongly influence ultrasound backscatter intensity, and it is correlated to GVF only when the bubble size distribution is known. The information on bubble size is difficult to obtain in field conditions causing this correlation to break down. Consequently, it is difficult to reliably apply methods based on ultrasound backscatter, and by extension its attenuation, for the quantification of GVF during influx events in a borehole. These methods can however be applied as highly sensitive detectors of gas bubbles for GVF $\ge$1 vol$\%$.

## Introduction

Drilling boreholes through the subsurface formation is necessary for extraction of hydrocarbons from subsurface oil and gas reservoirs, developing geothermal energy resources and, carbon capture and storage. The pressure of the drilling mud in the borehole is usually maintained at a slightly higher level compared to the formation pressure. However, some events during drilling may cause the borehole pressure to drop below the formation pressure, causing unintended influx of formation fluid into the borehole. Such events are called “kicks”, and early kick detection is often crucial for safe drilling operations. In a worst-case scenario, a delayed response to a kick may escalate into an uncontrolled release of hydrocarbon at the surface, known as a “blowout”.

Formation pressure and temperature generally increases with vertical depth, and for deep boreholes, values of 10,000 psi and 150 $^{\circ }$C are not uncommon. Under these conditions, influx of gas or gas-condensates exist in the supercritical state of matter, and thus their mass and density are closer to that of their liquid states. The influx gases expand in volume as they rise up the borehole, towards the surface, as the pressure and temperature drop below their critical points. Depending on the drilling depth, it can take several minutes for the influx gas to rise up to this point where it may become detectable in the drilling process parameters, if the quantity of gas in mud is sufficiently large. The low sensitivity of detection hampers the ability of well operators to initiate timely corrective action. Solubility of gas in the drilling mud further complicates this phenomenon^1–3^. In shallow gas drilling where the depth is typically < 1000 m, the influx gas invades the borehole directly in gaseous state and even a small mass of influx manifests as a large change in volume of the drilling mud in the borehole. Consequently, the time between actual influx and its manifestation on the surface is very short, which means that well operators have a very brief time window to carry out effective actions^4^. Therefore, early detection of gas influx into the drilling mud while drilling is of significant interest to the drilling operators from a safety and drilling efficiency perspective.

Current methods employed in the field for gas kick detection involve monitoring the volume flow rate of the return drilling mud, which will increase steadily if gas is present in the return mud. However, the sensitivity of this method is usually very low. Another common method involves mass flow measurement in the return mud line using Coriolis flow meters, but it requires that the mud line is fully loaded with gas for accurate measurement^5,6^, implying low sensitivity. Advanced mud logging using gas chromatography is also used for gas kick detection, amongst other things, by the compositional analysis of gas in the mud return line^7^. Recently, machine learning techniques have been developed for use on advanced mud gas logs for their rapid evaluation^8^. These methods however, are restricted to usage at the surface as they rely on sophisticated instrumentation for the analysis. This can lead to significant delay in information depending on the well depth, diameter and circulation rate of drilling mud.

**Figure 1:**
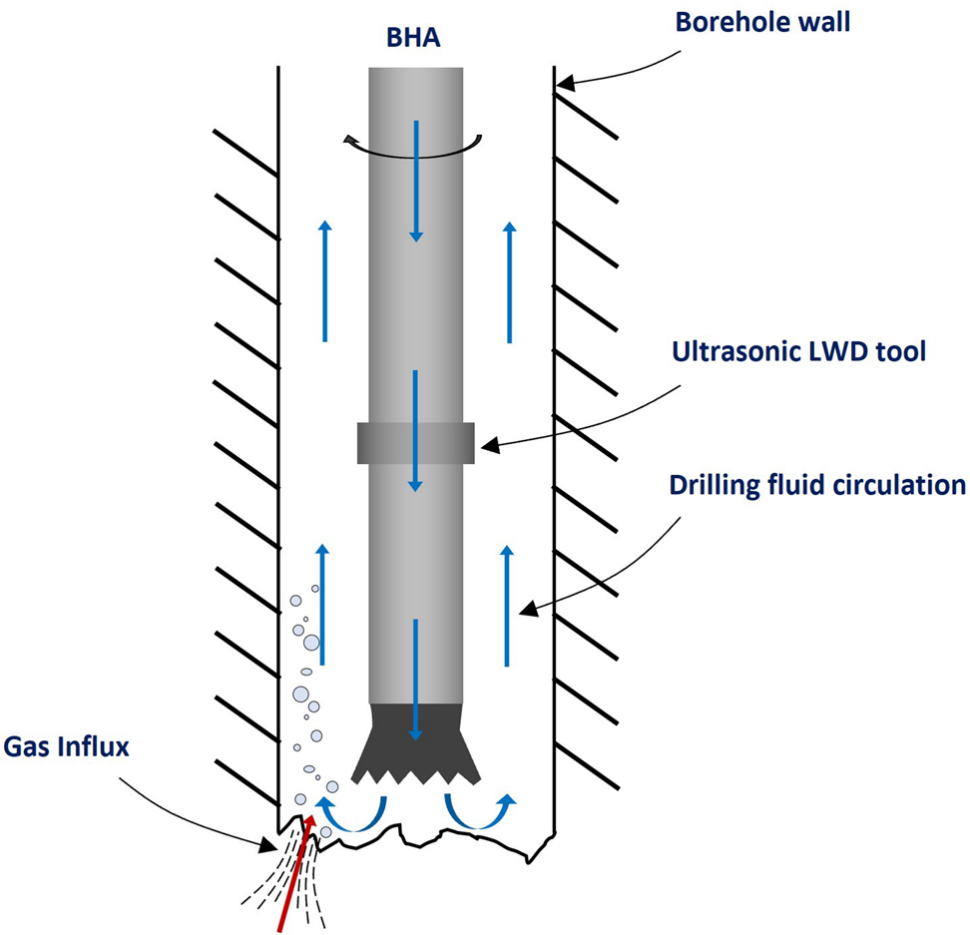
Schematic of a BHA with an ultrasonic LWD tool inside a borehole indicating a gas influx event.

The drilling operations are carefully monitored using various logging-while-drilling (LWD) tools^9^. Figure 1 is a schematic representation of a typical borehole assembly (BHA) with ultrasonic LWD tools. The drilling fluid is circulated from the surface, through the centre of the drill-string, through the BHA and drill bit nozzles into the borehole, carrying the rock cuttings back to the surface through the annulus between the borehole and the BHA. If the formation contains gas, this will be released into the drilling mud as the drilling progresses through that section of the formation, as depicted in Fig. 1. It would be highly beneficial if the existing ultrasonic LWD tools can also be used for the detection and quantification of such gas influx in the boreholes, during the drilling operation. This would eliminate the need for major modifications or increased complexity in the BHA.

Ultrasonic methods for detecting free gas in the drilling mud have been explored by several researchers. Most recently, monitoring the flow velocity of the drilling mud in the return line, at or close to the surface, using Doppler ultrasound has been explored^10–12^. These methods rely on an increase in volume of the drilling mud in the return line containing gas as it flows toward the surface due to the reduction in formation pressure and temperature. The increase in volume corresponds to an increase in flow velocity of the drilling mud. Consequently these methods are usually proposed to be applied around the riser section of a well, above the sea floor, where maximum increase in volume of gas is observed. The acoustic properties of multi-phase materials containing voids or gas has been extensively studied^13^ and used for estimating the bubble population^14^, void fraction and sound speed^15,16^ in such media. Hage et al.^17^ discuss a similar method based on a modified Wood’s equation for evaluating the velocity and phase change of an acoustic wave propagating along the annulus of the borehole. Adaptations of this principle are also evaluated numerically^18^ and, experimentally^19–25^.

The studies referred in the previous paragraph have suggested seemingly deterministic relationships between the ultrasonic response and gas volume fractions in the drilling mud under general operating conditions. Although these studies have considered many of the effects due to the complex interaction of ultrasonic waves in drilling mud with gas, the effect of bubble size distribution and its effects on the scattering of the ultrasonic wave field have not been considered. This aspect is very important as the bubble size in a gas influx event is usually unknown and may vary significantly, which in turn has a major nontrivial influence on the ultrasonic response. This paper discusses the scattering theories influencing this behaviour at different bubble sizes and combines them to form a simple generalised model which is analysed in the context of gas influx events in boreholes using simulations and laboratory experiments. The observations made in this paper are however broadly applicable to similar applications where the estimation of bubble populations in a fluid with unknown bubble size distributions is of interest.

The attenuation of bulk ultrasonic waves is generally considered to be caused by a combination of absorption and scattering^26^. Absorption is due to energy loss in the material due to its properties like viscosity^27^ and thermal conductivity^28^, which can be considered constant under known conditions of temperature and pressure. The energy loss due to scattering is the part of the incident acoustic energy not returning back to the transducer. This can be due to inhomogeneities within the material, causing local changes in acoustic impedance, within the parent material. Scattering can also occur due to the presence of a second phase within the material, including gas bubbles in context of kicks in boreholes.

The focus of this paper is to study the ultrasonic scattering in liquids containing bubble clouds with varying bubble sizes, in the context of its application while drilling boreholes. A simulation model is proposed, where the effect of ultrasonic backscatter from bubble clouds of any size distribution may be analyzed. The study also includes laboratory experiments where it is attempted to replicate bubble size distributions similar to what may occur during a borehole influx event. An ultrasonic transducer configuration similar to ones typically used in LWD is chosen in the context of its application for shallow gas blowout detection. However, the general principles discussed in this paper are applicable broadly to all similar transducers. In this study, water is used to emulate the drilling fluid, and air is used as the influx gas. The observations discussed in this paper however would apply to any gas–liquid combination in general. This work does not aim at replicating field conditions in the experimental setup or in the simulations, but rather to propose a reasonable framework to show that the ultrasound backscatter is strongly dependent on the bubble size distribution in the context of influx events in boreholes.

## Methodology

### Influx model and bubble size distribution

**Figure 2:**
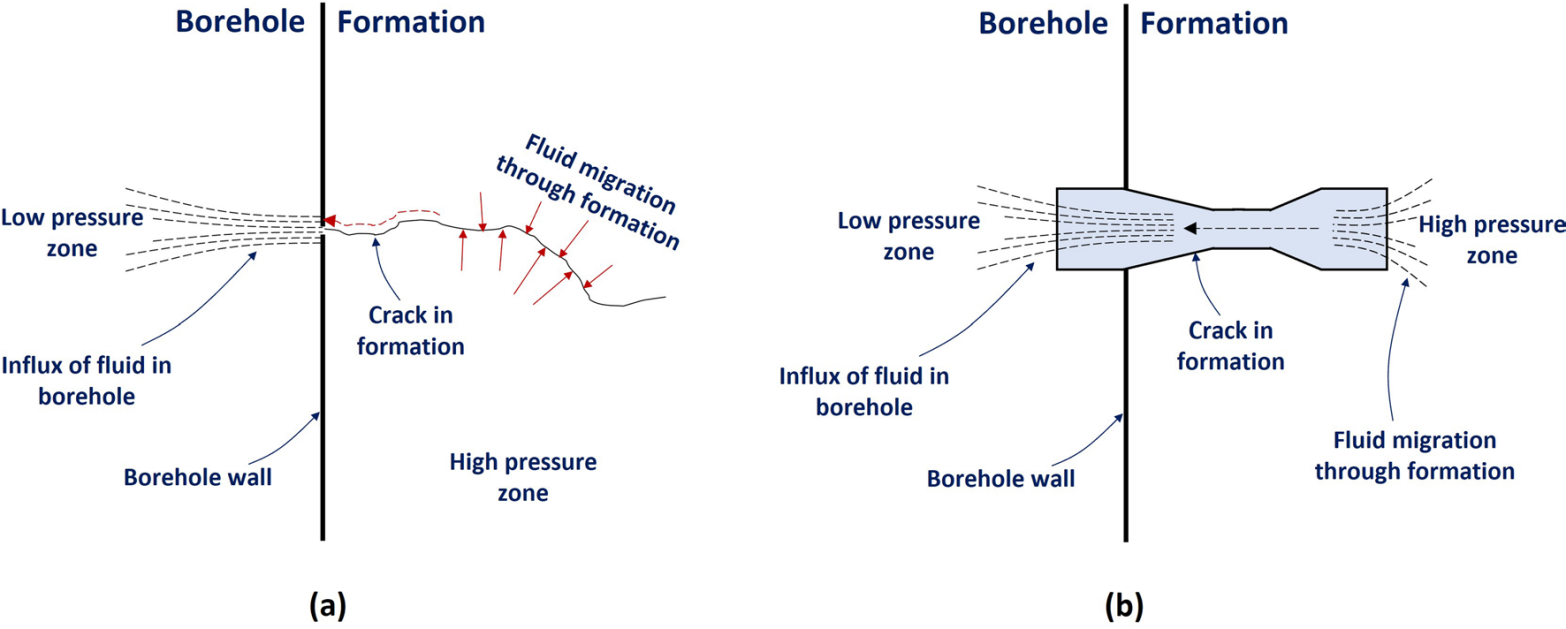
Schematic representation of, (**a**) fluid influx in a borehole through a crack in the formation and, (**b**) a model emulating such conditions as flow through a Venturi nozzle.

The movement of fluids through cracks and pores in the subsurface formation is well understood^29^. It is not unreasonable to expect a similar mechanism during fluid influx into the borehole from the formation. During an influx event, the formation pressure is at a higher level compared to the borehole. This pressure difference is the driving force for the fluid movement into the borehole through the crack/pore. The influx of fluid into the borehole through cracks/pores (Fig. 2a) may be modelled as the flow through a Venturi nozzle^30^ as depicted in Fig. 2b. The formation end of the crack behaves like a convergent nozzle, pulling fluid into it from a high pressure zone; while the borehole end of the crack behaves like a divergent nozzle, pushing fluid out into a low pressure zone. This model has been applied in the experimental setup used in this paper.

The exact bubble size distribution in borehole influx events are unknown to the best of the authors’ knowledge. The bubble size distribution may be further altered by various turbulence effects due to motion of the BHA. The proposed Venturi nozzle model is thus a crude approximation at best, and the bubble size distribution generated by such a Venturi nozzle may not exactly represent field conditions. Acknowledging these limitations, the proposed Venturi nozzle model remains a useful tool to understand the changes in size distribution of bubbles for different water flow rates. The ultrasonic backscatter for such changes can then be studied both experimentally and using the simulation model. It must be noted that the attempt is not to do a one-to-one comparison of the simulation model and the experiments, which is beyond the scope of this paper, but rather to study the dependence of ultrasound backscatter on bubble sizes both computationally and experimentally. A rough approximation of the bubble size distribution from a Venturi nozzle is sufficient for this purpose. The simulation model would then also allow us to study the ultrasonic backscatter on size distributions that are very different from that used in our study e.g. uniform bubble sizes, which is a common assumption in earlier studies discussed in “Introduction”.

The size distribution of bubbles generated by a Venturi nozzle has been studied experimentally^31,32^. An observation of results from these studies suggest that the bubble size distributions are similar to a subset of positive valued exponential distributions. It is possible to do a detailed statistical analysis of bubble sizes to select the most appropriate distribution. However, the Venturi nozzle model itself is a crude approximation of field conditions, and we only intend to study the dependence of ultrasound backscatter on bubble sizes in the context of influx events in boreholes, using experiments and simulations. Therefore, the specific choice of distribution within the family of positive valued exponential distributions should not affect the conclusions of this paper. We choose the Rayleigh distribution as a test case in our simulation model, where the bubble size distribution described by the random variable *A* has the probability density function

1 $$\begin{aligned} r(a;\beta )=\frac{a}{\beta ^2}e^{-a^2/2\beta ^2} \qquad a\ge 0, \end{aligned}$$

where $a$ is the bubble diameter and $\beta$ is the scale parameter. The choice of using the Rayleigh distribution for bubble sizes allows us to control the bubble size distribution with a single parameter $\beta$, known as the scale parameter. The scale parameter $\beta$, of the Rayleigh distribution is a measure of its standard deviation which can by definition be expressed as

2 $$\begin{aligned} std(A)=\beta \sqrt{(2-\frac{\pi }{2})} \approx 0.655\beta , \end{aligned}$$

where $std(A)$ is the standard deviation of the random variable A in Eq. (1).

Fujiwara et al.^32^ show experimentally that the histograms of bubble sizes generated by a Venturi nozzle are wider for lower throat velocities in the nozzle, and become narrower as the throat velocity is increased. This is analogous to an inverse relationship of the water flow velocity through the Venturi nozzle to the standard deviation of the histogram of bubble sizes and by extension the scale parameter $\beta$ of the Rayleigh distribution. We could make similar observations through size analysis of bubbles generated from the Venturi nozzle used in our experiments as discussed later in this paper (Fig. 6). Further, Murai et al.^33^ also show experimentally that the mean size of bubbles generated by a Venturi nozzle is proportional to the initial bubble size injected at the inlet of the Venturi nozzle. The size of the bubble injected at the inlet of the Venturi nozzle is equivalent to its volume, and the mean bubble size produced by the Venturi nozzle is analogous to the mean of the Rayleigh distribution, which by definition is related to the scale parameter $\beta$, as

3 $$\begin{aligned} \mu (A)=\beta \sqrt{\frac{\pi }{2}} \approx 1.253\beta , \end{aligned}$$

where $\mu (A)$ is the expectation value of *A* in Eq. (1). Thus, the scale parameter $\beta$, can be said to be proportional to the volume of air injected at the throat of the Venturi nozzle and inversely proportional to the water flow velocity through the Venturi nozzle.

### Simulation setup

The behavior of gas bubbles in a liquid, under an ultrasonic field, differs from that of rigid spheres depending on the physical properties of the gas and the liquid. The gas enclosed within the bubble provides a stiffness to the ultrasonic field causing it to oscillate radially, like a spring. The fluid surrounding the bubble in turn resists the oscillation due to its inertia^34^. These effects, in addition to the physical properties of the gas and the liquid, are also a function of the frequency of the ultrasonic field and the bubble size. Any simulation model must therefore incorporate these effects. This could be achieved e.g. by using a multi-physics model combing computational fluid dynamics (CFD), for capturing the bubble dynamics, with wave propagation modelling for capturing the ultrasonic field response. Commercial software like COMSOL, ABACUS etc. could be used for this purpose, but would still require considerable knowledge in CFD and wave propagation to model these complex phenomenon. Instead, we propose and use a simpler approach for these simulations using the ultrasound simulation program—Field II^35,36^.

Field II requires the imaged objects to be defined as a collection of point scatterers in space. The spacial and temporal ultrasonic field from each scatterer is evaluated and summed to obtain the received signal. For our simulations, modelling the size of each bubble is important as the scattering from each bubble is a function of its size^34^. The surface of each bubble can thus be emulated by creating several point scatterers about a sphere and sufficiently close together ($\ll$wavelength), such that their reflected ultrasonic fields completely overlap and thus become unresolvable. Further, the radial oscillation of the bubbles due to the ultrasonic field must also be modelled. This can be achieved in principle by calculating the temporal position of every point scatterer on the bubble surface due to the oscillations using first principles. Executing such a model would be a very complex task and considering that each simulation would contain > 500 bubbles on average, this approach would require a model containing several tens of thousands of point scatterers. The computation time for each simulation would then be very high. Further, a large number of simulations are required for various gas volume fractions (GVF) and bubble sizes, with each set of conditions to be repeated several times for statistical soundness. Consequently, the total simulation time would be prohibitively high. We utilize an alternative strategy to significantly reduce the model complexity. Instead of geometrically arranging several point scatterers to emulate a single bubble, we model every bubble as a single scatterer and modify their reflection coefficients according to their size. Field II utilizes the Born approximation wherein the effects due to the scatterers’ position relative to each other are not considered. The bubble cloud in the simulation model is in the form of a 10 mm thick slice at distance of 30 mm from the transducer wherein the bubbles are uniformly distributed in space. The scattering of bubbles much smaller than the wavelength can be described as Rayleigh scattering, and this can be modelled as described by Medwin et al.^34^,

4 $$\begin{aligned} \sigma _s^R = \frac{4\pi a^2}{[(f_R/f)^2-1]^2+\delta ^2}, \end{aligned}$$

where $\sigma _s^R$ is the scattering cross-section for a bubble of radius $a$, $f_R$ is the resonance frequency of the bubble, $f$ is the frequency of ultrasonic wave, and $\delta$ is the damping constant which accounts for viscoelastic effects of the bubble under the influence of the ultrasound field.

The scattered ultrasonic wave field propagates spherically in all directions from the scatterer. Thus, the response as recorded by the ultrasonic transducer is approximately the fraction proportional to the angle subtended by the scatterer on the transducer. This is called the backscatter, and thus from Eq. (4), the backscattering cross-section per steradian $\sigma _{bs}^R$ takes the form,

5 $$\begin{aligned} \sigma _{bs}^R = \sigma _s^R/4\pi . \end{aligned}$$

The backscattered intensity per steradian can then be expressed as,

6 $$\begin{aligned} I_{bs}^R = I_0\times \sigma _{bs}^R = I_0\frac{\sigma _s^R}{4\pi }, \end{aligned}$$

where $I_0$ is the intensity of the incident ultrasonic wave.

Further, the scattering from larger bubbles can be considered as specular reflection. For gas bubbles in a liquid, the acoustic impedance missmatch is large, and we can then assume perfect reflection of the ultrasonic wave. The backscattering intensity is then described by Hunter et al.^37^,

7 $$\begin{aligned} I_{s}^S = I_0\frac{a^2}{(2d - a)^2}, \end{aligned}$$

where $d$ is the distance between the transducer and bubble. For the conditions discussed in this paper, this distance is between 30–40 mm. This is very large compared to the radius ($a$) of individual bubbles (generally <500 $\upmu$m), and thus $(2d-a)\approx 2d$. The specular backscattering intensity at the transducer surface per steradian can then be approximated from Eq. (7) as,

8 $$\begin{aligned} I_{bs}^S = I_s^S\times d^2 \approx I_0\frac{a^2}{4}. \end{aligned}$$

**Figure 3:**
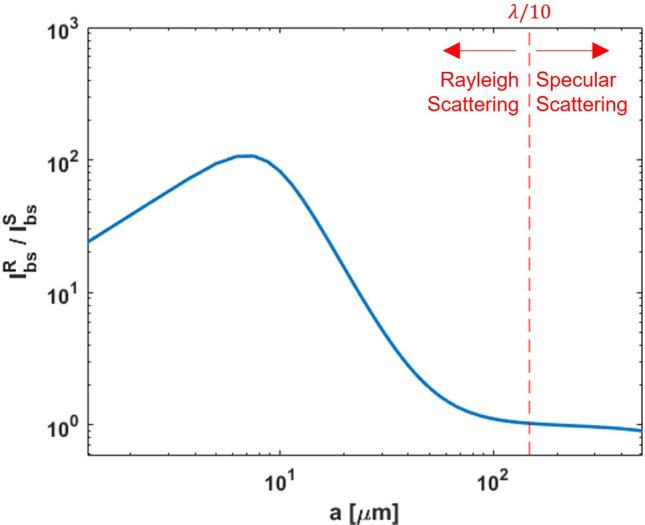
Ratio of backscattering intensity due to Rayleigh scattering and specular reflection versus bubble size at ambient conditions for air bubbles in water.

**Figure 4:**
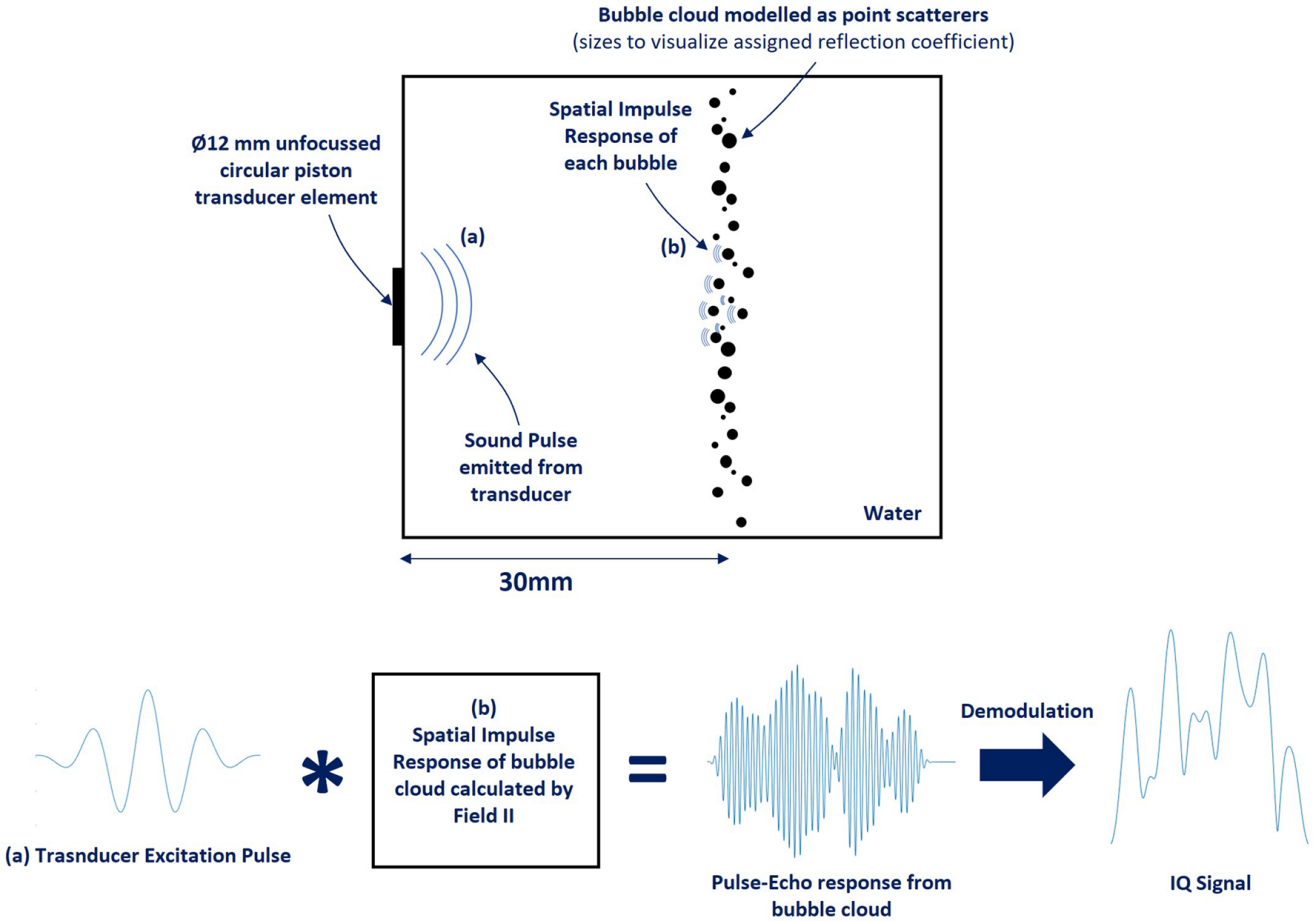
The simulation setup implemented in Field II depicting the signals at key processing steps to obtain the IQ signal from the bubble cloud.

The contributions of Eqs. (6) and (8) versus the bubble size is clearly seen in Fig. 3, for air bubbles in water at ambient conditions. The ratio $I_{bs}^R/I_{bs}^S$ approaches 1 at $a \approx \lambda /10$, suggesting that Eq. (6) dominates for $a <\lambda /10$, while Eq. (8) dominates for larger bubble sizes. Thus, we can use the $a \approx \lambda /10$ limit for classifying bubbles into two size groups; and applying Eq. (6) for the group with $a <\lambda /10$, and Eq. (8) for the group with $a >=\lambda /10$. The peak observed at $a=$7.52 $\mu$m, in Fig. 3 corresponds to the resonance frequency of the bubble ($f_R$ in Eq. (4)), also called as the Minnaert frequency. It must be noted that these values would change for downhole conditions in a borehole, but the general principles discussed in this paper would still be valid. This would also hold for other applications with different fluid combinations and working conditions within the scope of assumptions made herein.

We use the back-scattering intensity as evaluated from Eqs. (6) and (8) as the reflection coefficient of the point scatterers in Field II depending on the bubble size. The size of the bubbles $a$, is modelled using a Rayleigh distribution (Eq. 1). Field II evaluates the spatial impulse response from the bubbles in the model which is convolved with the transducer excitation pulse to obtain the pulse-echo response from the bubble cloud. The evaluation is done for a Ø12 mm unfocused circular piston transducer element with a 50% bandwidth at 1 MHz. The excitation pulse consists of a 4-cycle Gaussian modulated sine pulse. The sound speed of the medium around the transducer element is set to 1480 m s^−1^ to emulate water. The effects of absorption loss in the medium is neglected as the intention is to study the effect of scattering alone. The pulse-echo response is demodulated to get the in-phase quadrature (IQ) signal. The power of the IQ signal is used as a measure of the cumulative backscatter intensity $I$, from the bubble cloud. The simulation setup is depicted in Fig. 4

### Experimental setup

**Figure 5:**
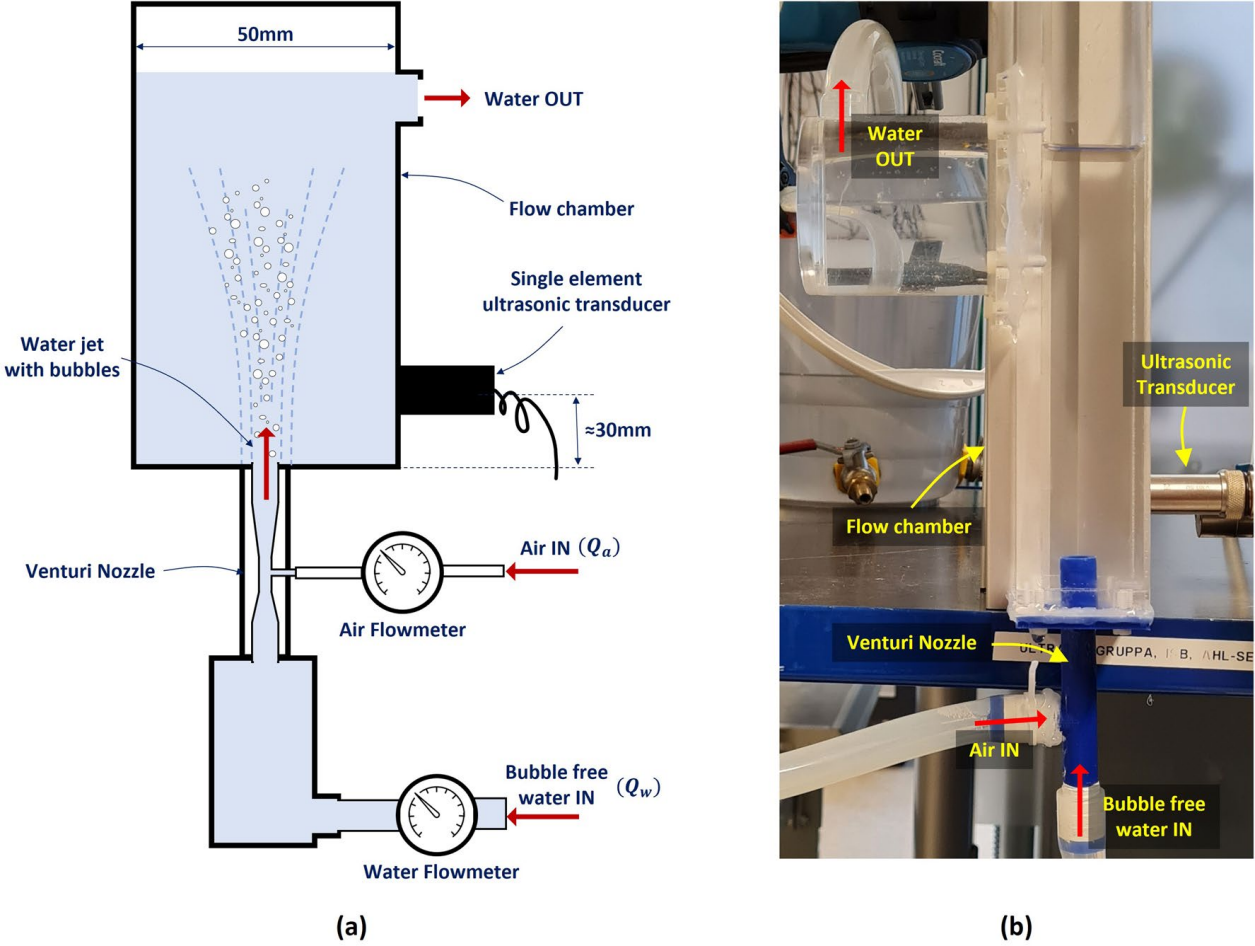
Experimental setup used for generating bubble clouds in water using a Venturi nozzle and a single element ultrasonic transducer for imaging the bubble cloud. (**a**) Schematic representation of the setup and, (**b**) a photograph showing the flow cell and Venturi nozzle.

The experimental setup consists of a vertical flow chamber, made of plexiglas, as shown in Fig. 5. The width of the flow chamber is 50 mm, which is similar to the typical annular spacing between the BHA and borehole wall. A Venturi nozzle is attached at the inlet end of the flow chamber for creating bubbles as described earlier.

The design of the Venturi nozzle was inspired from Wiraputra et al.^38^ and consists of a convergent part through which bubble-free water is pumped in ($Q_w$). The water accelerates through the convergent part of the nozzle with a corresponding pressure drop. The pressure drop is maximum at the throat of the convergent part of the nozzle. Controlled amounts of air ($Q_a$) can be injected into the throat of the nozzle through an air inlet provision at this location. A divergent part then follows the throat of the convergent part of the Venturi nozzle, where the pressure gradually rises with corresponding velocity drop. The increase in pressure in this region causes the air introduced at the throat to collapse and disintegrate into several small bubbles^32^, which flow out of the Venturi nozzle and into the flow chamber. The Venturi nozzle used for the experiments in this paper was 3D printed and can be seen in Fig. 5b.

**Figure 6:**
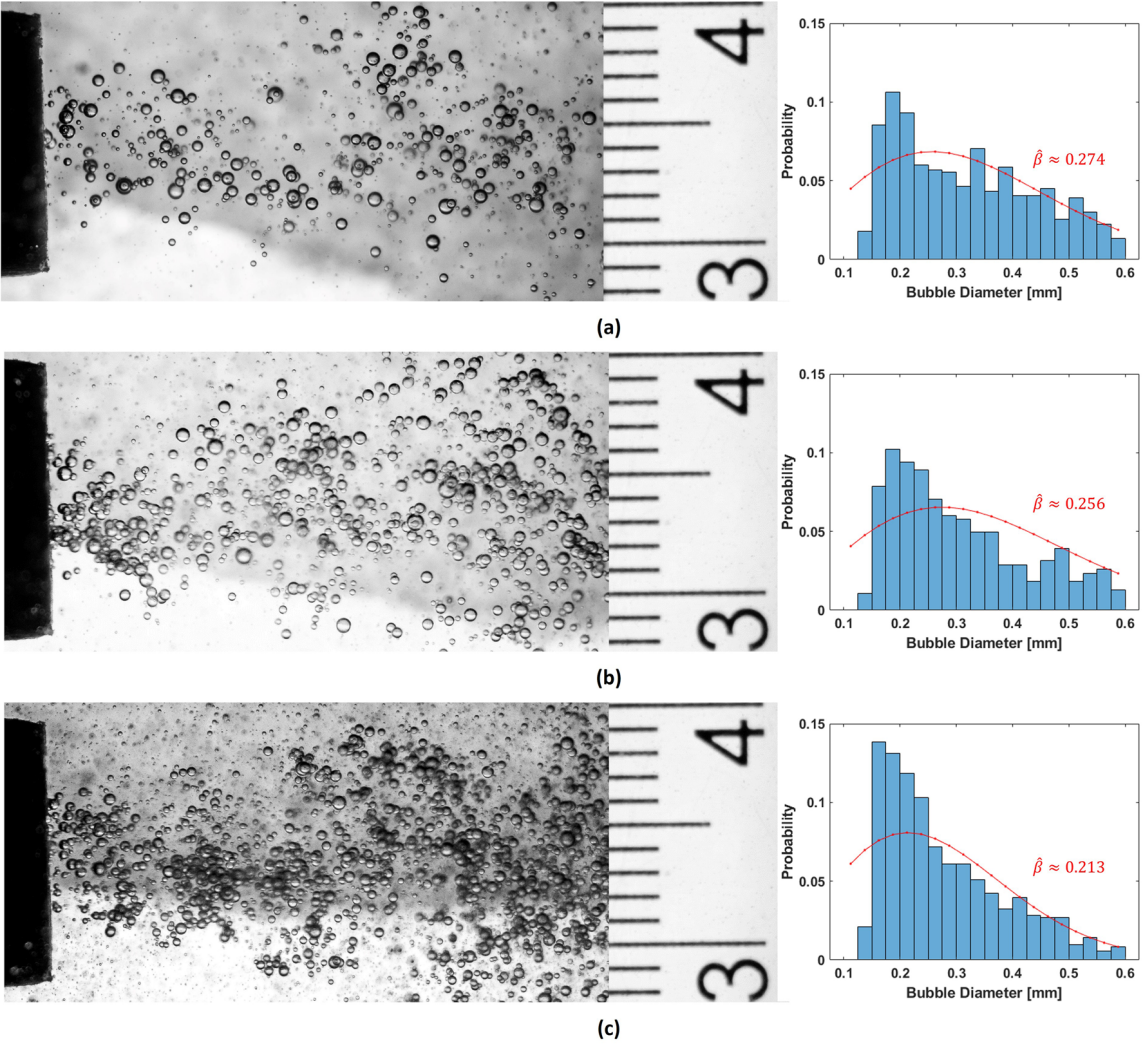
Photographs of bubbles from the Venturi nozzle and their size distribution histograms, at water flow rates of (**a**) 1 lpm, (**b**) 1.5 lpm, (**c**) 2 lpm; and constant air influx rate of 0.04 lpm. The red overlay curves on the histograms are Rayleigh distribution fits with 95% confidence level and the maximum likelihood estimates of the scale parameter $\hat{\beta }$.

We have discussed earlier the inverse proportionality of bubble sizes from a Venturi nozzle to the scale parameter $\beta$ of a Rayleigh distribution, based on experimental observations by Fujiwara et al.^32^. To ascertain that bubbles produced by our 3D printed Venturi nozzle also have a similar behaviour, we captured images of the bubbles from the Venturi nozzle for different water flow rates $Q_w$ through the Venturi nozzle with a constant air injection rate at the throat $Q_a$= 0.04 lpm. The photographs of the bubbles were captured with a high shutter speed of 1/8000 s, using a Canon EOS70D camera mounted with a Canon Zoom lens EF-S 18–135 mm. The focal plane of the camera was adjusted approximately to the centerline of the Venturi nozzle. A millimeter scale was also photographed separately at exactly the same focal distance used for capturing the bubbles. The photographs of the bubbles with the millimeter scale adjacent to it are shown in Fig. 6. The imageSegmenter tool from Matlab Image Processing Toolbox (2022b, The Mathworks, Natick, MA, USA) was used to segment the bubbles in the images as circles and their diameters are extracted in terms of number of pixels. This can be converted into millimeters by comparing it with the number of pixels between two scale lines in the image of the millimeter scale (for the image shown in Fig. 6: 76 pixels = 1 mm). It must be noted that the segmentation depends on detecting the dark edges of the bubbles in the image. It must be also noted that only the bubbles very close to the focal plane of the camera would be captured clearly and have well defined edges and all bubbles out of focus would be blurred out. Overlapping bubbles would also cause the bubble edges to blur out. These effects would cause errors in segmentation of bubbles in the imaging plane. It is difficult to quantify these errors or eliminate them with the existing instrumentation. Any attempt to accurately fit a specific distribution on this data will be quite difficult. However, considering that we are using a Rayleigh distribution as a test case for our simulation model, it is useful to observe the changes in the Rayleigh distribution fits for bubble sizes in these images. Histograms of bubble sizes from the images are shown in on right hand side of the respective photographs in Fig. 6. The red overlay curves on the histograms are Rayleigh distribution fits with 95% confidence level and the maximum likelihood estimates of the scale parameter $\hat{\beta }$. There are clear discrepancies between the Rayleigh distribution fit and the histogram, suggesting that the Rayleigh distribution is only an approximate fit. It can however be observed that the scale parameter $\hat{\beta }$ of the Rayleigh distribution fits vary inversely to the water flow rates $Q_w$ through the Venturi nozzle.

It is standard practice to specify the amount of gas influx in drilling mud in terms of gas volume fraction (GVF),

9 $$\begin{aligned} GVF=\frac{V_g}{V}, \end{aligned}$$

where $V_g$ is the volume of gas and, $V$ is the total volume of all fluids in the borehole. This study covers a GVF range of 1–40 vol% in the simulations and experiments. The GVF in the experimental setup can be controlled by adjusting the water and air inflow rates through the Venturi nozzle as per Eq. (9),

10 $$\begin{aligned} GVF=\frac{V_g}{V}=\frac{Q_a}{Q_w+Q_a} \qquad \because Q=A\times V. \end{aligned}$$

Flowmeters are used at the air and water inlets to help regulate the respective flows.

A Ø12 mm 1 MHz single element transducer similar to those used on the BHA for LWD, is used for all experiments and simulations in a pulse-echo configuration (see Indimath et al.^39^ for a detailed discussion on transducer selection). The back-scatter intensity from the bubble cloud flowing in water is evaluated. The overflow of bubbly water in the flow chamber is discarded continuously to ensure that there is no build-up of residual bubbles in the flow chamber.

## Results and discussion

### Uniform bubble sizes

**Figure 7:**
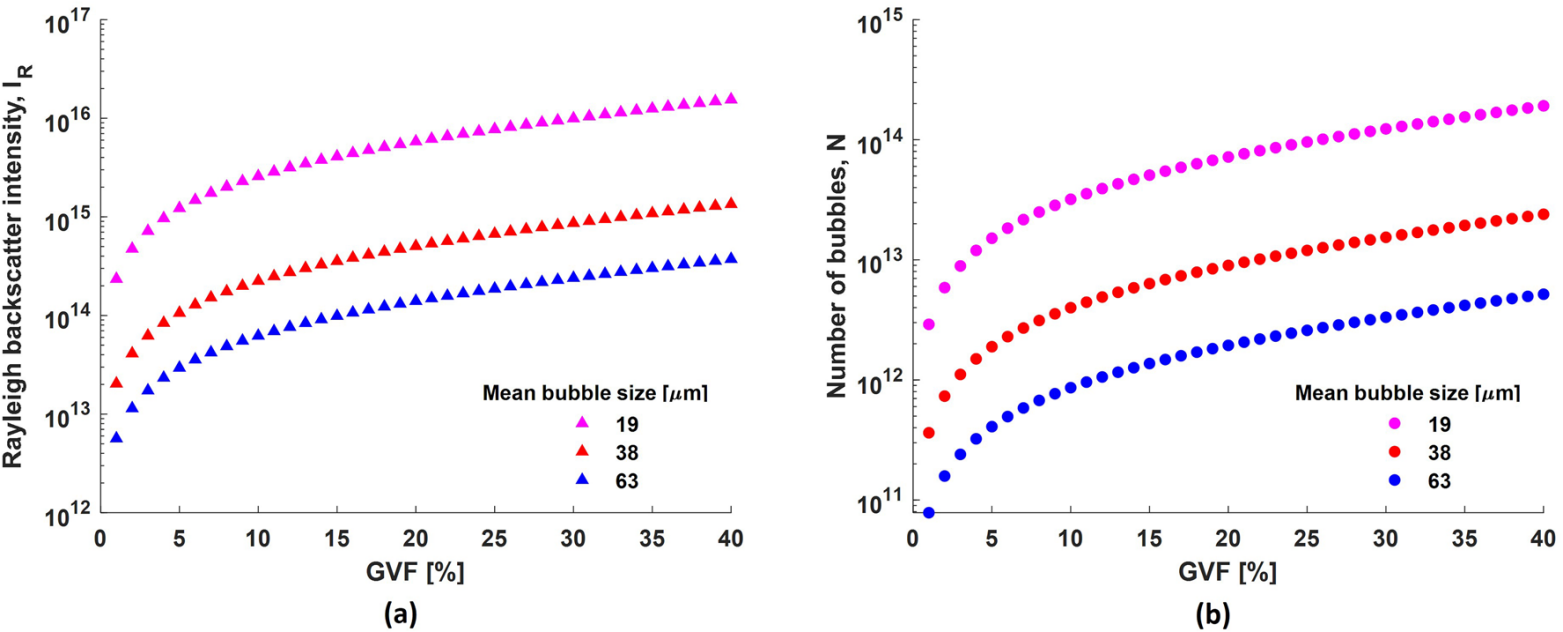
(**a**) Simulated Rayleigh backscatter intensity from clouds of uniformly sized bubbles for different GVF and, (**b**) the corresponding number of bubbles in the bubble cloud.

**Figure 8:**
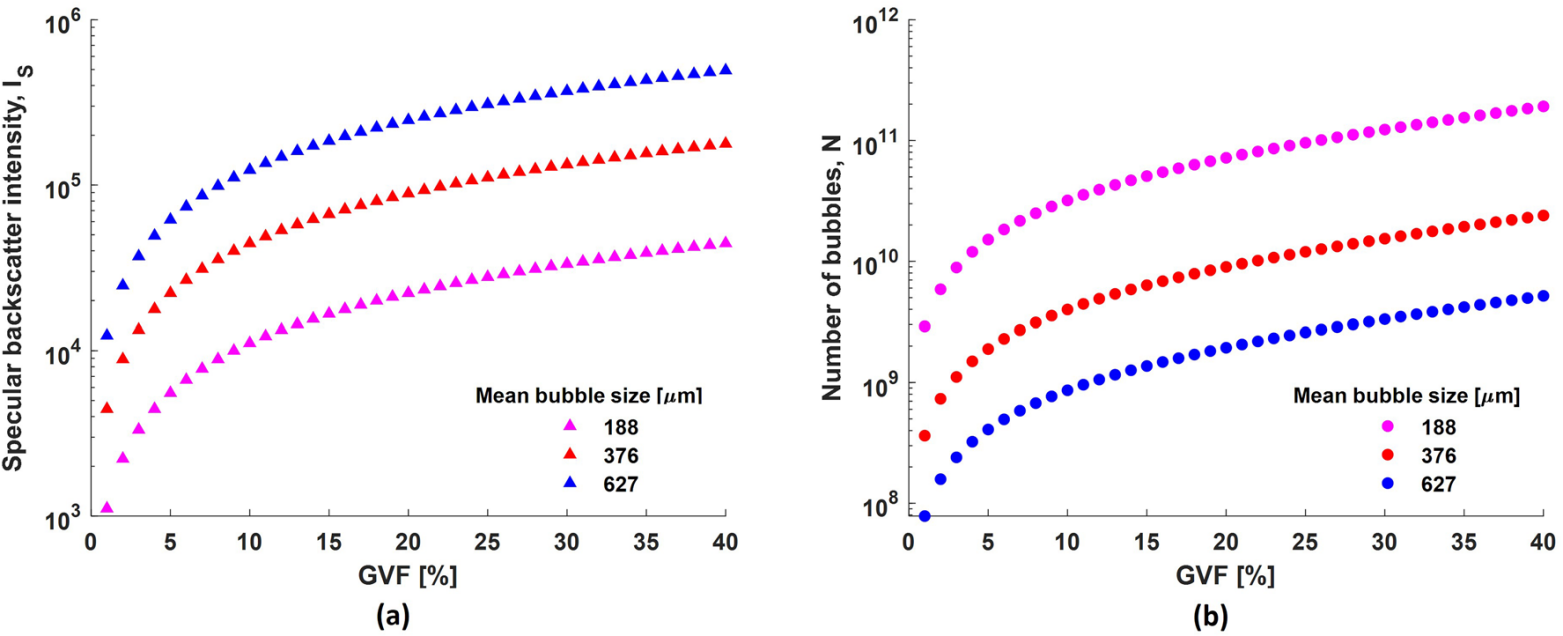
(**a**) Simulated specular backscatter intensity from clouds of uniformly sized bubbles for different GVF and, (**b**) the corresponding number of bubbles in the bubble cloud.

This section discusses the behaviour when the size of all bubbles is considered constant. This condition is similar to the assumption in most previous studies discussed in the introduction. GVF as defined earlier, is the volumetric ratio of gas in the liquid. In the present context, the gas (air) is present in the form of bubbles in the liquid (water). Consequently, GVF is a function of the total number of bubbles $N$, and the volume occupied by each bubble. The volume of the bubble is in turn a function of the third power of its diameter $a$. Thus, the total number of bubbles for a particular GVF is inversely proportional to $a^3$ (Figs. 7b,  8b),

11 $$\begin{aligned} N \propto \frac{1}{a^3} \end{aligned}$$

The nature of ultrasound backscatter from the bubbles depends on the bubble size as described earlier. Accordingly, simulations were performed in both regimes using the scattering cross sections from Eqs. (5) and (8) for different bubble sizes and GVF, and the results are shown in Figs. 7a and 8a.

It is observed that the backscatter intensity in both regimes is correlated to the GVF when viewed for a specific bubble size. However, it is worth noting that the specular backscatter intensity displays an inverse relationship to the bubble size (Fig. 8), whereas the Rayleigh backscatter intensity displays a direct relationship (Fig. 7). This result is not intuitive and can be explained as follows. The total backscatter intensity from a bubble cloud is proportional to the sum of backscatter intensities from each bubble insonified by the ultrasound beam. The Rayleigh backscatter intensity from a single bubble is proportional to the sixth power of bubble size ((Eq. 6): $\because \delta \propto 1/a^2$ and $f_R \propto 1/a$ in Eq. (4)^34^) while, the specular backscatter intensity is proportional to the second power of bubble size (Eq. 8). Thus, the total backscatter intensity ($I_{tot}^{R}$) from a bubble cloud containing $N$ uniformly sized bubbles in the Rayleigh regime,

12 $$\begin{aligned} I_{tot}^{R} \propto N\times I_{bs}^R \propto a^3 \qquad \; \because \; I_{bs}^R \propto a^6 \end{aligned}$$

while the total backscatter intensity ($I_{tot}^{S}$) from a bubble cloud containing $N$ uniformly sized bubbles in the specular regime,

13 $$\begin{aligned} I_{tot}^{S} \propto N\times I_{bs}^S \propto \frac{1}{a} \qquad \quad \because \; I_{bs}^S \propto a^2 \end{aligned}$$

It was not possible to investigate these conditions experimentally as it was not possible to generate a bubble cloud with equally sized bubbles using our experimental setup. Nevertheless, it can be inferred that the GVF in general has a good correlation to the ultrasound backscatter intensity, irrespective of the regime, if the bubble size is known and it remains constant throughout. It is unrealistic to expect such conditions in practical situations.

### Rayleigh distributed bubble sizes with constant scale parameter, $\beta$

We can bring the conditions nearer to practical conditions by eliminating the assumption of constant bubble sizes. Instead, we use a Rayleigh distribution for the bubbles size, which is relevant in the context of gas influx in boreholes. However, for each set of simulations and experiments, we choose to fix the scale parameter $\beta$, to a specific value as the GVF is increased. Additionally, we consider the fact that the bubbles can have a size distribution spanning both scattering regimes simultaneously. Using Eqs. (12) and (13), the backscatter intensity for each bubble $k$ in the model, can be expressed as,

14 $$\begin{aligned} I_k = {\left\{ \begin{array}{ll} I_{bs}^{R}(a_k) \qquad \text {for} \quad {a_k < \frac{\lambda }{10}} \\ I_{bs}^{S}(a_k) \qquad \text {for} \quad {a_k \ge \frac{\lambda }{10}} \end{array}\right. } \end{aligned}$$

where $a_k$ is Rayleigh distributed as per Eq. (1). These are conditions that may exist in a borehole during an influx event if the bubble size distribution can be estimated by an independent method. There is no such method in use at present to the best of the authors’ knowledge. Such conditions are also known to exist in other practical applications e.g. medical imaging using contrast agents.

**Figure 9:**
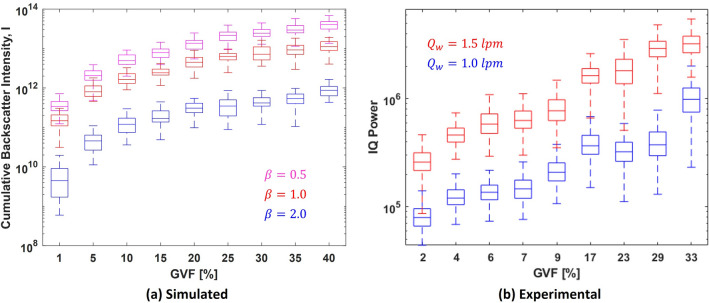
Backscatter intensity from a cloud of Rayleigh distributed bubble sizes with constant scale parameter $\beta$ from (**a**) simulations, where each box in the plot corresponds to 30 simulations and, (**b**) experimental, where each box in the plot corresponds to 200 IQ signals.

We perform the simulations using Rayleigh distributed bubbles with scale parameters $\beta$ equal to 0.5, 1 and 2, for GVF ranging from 1 to 40%. The expectation values of bubble diameter for the aforementioned values of $\beta$ are 0.63 mm, 1.25 mm and 2.5 mm respectively, as evaluated using Eq. 3. The results from 30 simulations for each $\beta$ and GVF combination is shown in Fig. 9a.

The results seem similar to those observed for purely Rayleigh scattering (Fig. 7a), even though both scattering regimes have been incorporated. This suggests that Rayleigh scattering is dominant even though the relative number of bubbles in this regime may be much smaller compared to those in the specular regime, especially for larger $\beta$ values. The relationship of backscatter intensity and $\beta$ would however be expected to invert for very large values of $\beta$, when the backscatter is predominantly specular.

Experimental evaluation using these conditions also display a similar result as shown in Fig. 9b. The scale parameter $\beta$ is adjusted by altering the water flow rate $Q_w$. We arbitrarily choose the values of $Q_w$ as 1 lpm and 1.5 lpm for the experiments. Recall that a higher $Q_w$ corresponds to a smaller $\beta$. Thus even with a realistic bubble size distribution, there is a good correlation between GVF and ultrasound backscatter intensity and by extension the attenuation. Knowledge of the bubble size distribution i.e. the mean and variance, reflected by the scale parameter $\beta$ is still a necessity.

### Rayleigh distributed bubble sizes with random scale parameter, $\beta$

Information about the bubble size (reflected by the scale parameter $\beta$) in a borehole is extremely difficult to obtain, and to the best of the authors’ knowledge, no method is in use thus far. Thus, to bring our study even closer to field conditions, we remove the assumption of a known scale parameter $\beta$. Instead, we let $\beta$ follow a uniform random distribution,

15 $$\begin{aligned} u(\beta ) = {\left\{ \begin{array}{ll} \frac{1}{\beta _{max}-\beta _{min}} \qquad \text {for} \quad \beta _{min}<\beta <\beta _{max} \\ \\ \qquad \; 0 \; \qquad \qquad \text {otherwise.} \end{array}\right. } \end{aligned}$$

**Figure 10:**
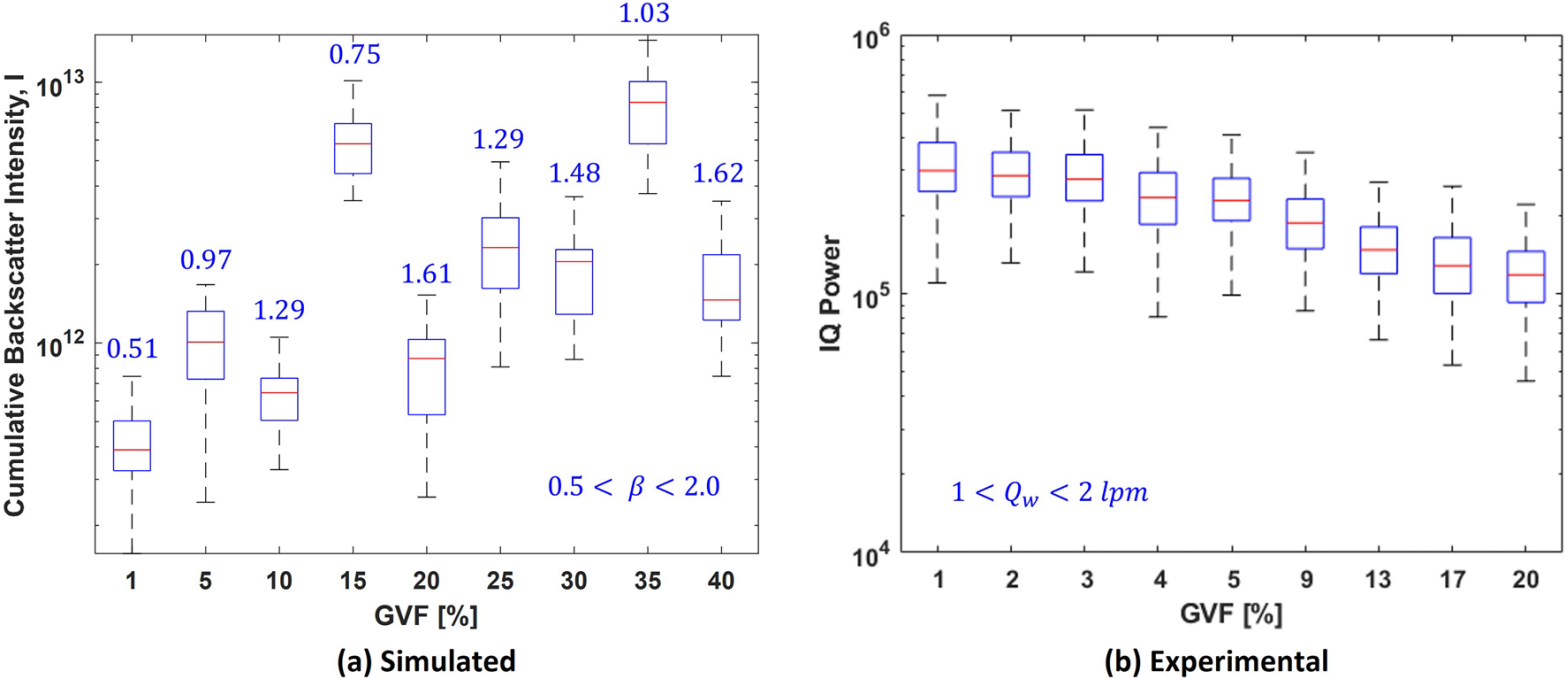
Backscattter from a cloud of Rayleigh distributed bubble sizes with randomized scale parameter $\beta$. Results are shown for (**a**) simulations where, each box in the plot corresponds to 30 simulations and values in blue colour above each box is $\beta$ value used for the corresponding set of simulations and, (**b**) experiments where, each box in the plot correspond to 200 IQ signals.

Thus, during an influx event in a borehole, assuming that $\beta$ changes randomly as the drilling progresses and consequently GVF increases steadily: we can use a random value of $\beta$ for each GVF arbitrarily between $\beta _{min} = 0.5$ and $\beta _{max} = 2$ as per Eq. (15) for the simulations. For the experiments, this is done by randomly setting the water flow rate $Q_w$ between 1 lpm and 2 lpm. The cumulative backscatter intensity for this case is the same as Eq. (14), but with $\beta \in u(\beta )$ as per Equation (15). The results from the simulations is shown in Fig. 10a and those from the experiments is shown in Fig. 10b.

It is evident from the results that the correlation between GVF and backscatter intensity no longer exists. The breakdown in correlation is more evident in the simulation results as the randomization of the scale parameter $\beta$ could be acheived with a much finer resolution between the set limits of 0.5 and 2. This was much difficult to achieve in the experiments due to the range of flow rates being restricted to a narrow window between 1 and 2 lpm. This is because for flow rates below 1 lpm, the stream of bubbles from the Venturi nozzle was intermittent rather than the desired continuous flow. With an intermittent bubble cloud, it was very difficult to time the ultrasound acquisition such that the entire cloud is within the ultrasound beam. Further, for flow rates larger than 2 lpm, the number of bubbles in the flow chamber was too high and could not keep pace with the gravity dependent extraction rate of the bubbly water. Thus bubbles from the top of the chamber would swirl back and mix with newly injected bubbles. This condition was not suitable for our study.

## Conclusion

The size distribution of bubbles in case of a gas influx in boreholes can vary significantly. The effect of variance in bubble sizes at different gas volume fractions (GVF), on ultrasound backscatter and by extension on the attenuation of ultrasound from bubble clouds has been presented. A model combining the Rayleigh scattering and specular reflection theories has been discussed and used with the ultrasonic simulation tool—Field II, and the results have been discussed in the context of its application in a borehole gas influx event. Experimental results with similar conditions as the simulations are shown to be in agreement with one another. Important observations derived from this study are as follows:

- The number of bubbles can vary significantly, depending on the bubble size distribution, for the same GVF. This results in an inverse relationship with bubble size for specular backscatter intensity and a third power dependence with bubble size for Rayleigh backscatter intensity versus the total number of bubbles insonified by the ultrasound beam.
- For applications where the bubble size distribution would span across both the Rayleigh and specular backscatter regimes; the Rayleigh scattering regime dominates the backscatter intensity even when the total number of bubbles in the Rayleigh scattering regime is smaller relative to those in the specular regime. The specular regime however, is expected to dominate once the bubble sizes in the population are predominantly larger than the wavelength. Irrespective of the scattering regime, a good correlation between ultrasound backscatter intensity and GVF is observed. This suggests that, in the presence of an independent estimate of bubble size distribution, GVF can be reasonably predicted using ultrasonic backscatter intensity, and by extension its attenuation.
- In a borehole influx setting, the exact bubble size distribution is very difficult to ascertain due to its dependence on various parameters like crack/pore size, borehole and formation pressure, etc. In the absence of this information, the correlation between ultrasonic backscatter intensity and GVF is observed to break down. Thus, ultrasonic methods relying on backscatter and by extension attenuation can be used for detection of gas in the drilling mud, but may be unreliable for its quantification. It is important to note that the detection sensitivity for GVF using ultrasonic backscatter is very high even for GVF $\ge$ 1 vol%.

Finally, it is important to note the known limitations of the studies presented in this paper.

- The bubble size distribution from a Venturi nozzle is assumed to be similar to a Rayleigh distribution. Although this does not affect the conclutions made in this paper, a thorough statistical study of bubble size distributions in field conditions during a borehole influx can be done in future studies. These size distributions can then be used in the proposed model to gain more insight for field implementation of this method.
- The simulations in this paper are done using the ultrasound simulation program—Field II, which utilizes the Born approximation for evaluating the ultrasonic field from the bubble cloud. Any errors caused due to this approximation, especially for larger GVF values are not accounted for in our model. The effects on the ultrasonic field due to the position of bubbles relative to each other are also neglected in this study as a consequence of the Born approximation.
- Two scattering theories viz. Rayleigh scattering for bubble size $<\lambda /10$, and specular scattering for bubble size $>\lambda /10$ are used in our model. However, it is not common to observe specular scattering unless the bubble sizes are $>\lambda$. This does not seem to significantly affect the observations made in this paper, considering that we observe very similar results in both simulations and experiments. Including another theory in the model e.g. Mie scattering for bubble sizes between $\lambda /10$ and $<\lambda$ could increase the accuracy of the model for certain applications.
- A systematic validation of the proposed simulation model has not been done. The authors propose this to be done in a future study to find gaps and enable further refinement of the model.

## Data Availability

Raw experimental data, associated processing algorithms and Field II simulation codes used can be made available upon request to Shivanandan Indimath at indimath.shivanandan@gmail.com.
